# P-811. Telavancin for Refractory MRSA Bacteremia: A 10-Year Retrospective Review

**DOI:** 10.1093/ofid/ofae631.1003

**Published:** 2025-01-29

**Authors:** Caroline Gander, Santhi Singanamala, Abriana Holzworth, Caleb Rux, Dayna McManus, Matthew W Davis, Jeffrey E Topal

**Affiliations:** Community Health Network, Indianapolis, Indiana; Trinity Health of New England, Hartford, Connecticut; Yale New Haven Hospital, New Haven, Connecticut; Loyola University Medical Center, Maywood, Illinois; Yale New Haven Hospital, New Haven, Connecticut; Yale New Haven Hospital, New Haven, Connecticut; Yale New Haven HospitalYale University School of Medicine, New Haven, Connecticut

## Abstract

**Background:**

Refractory methicillin-resistant *Staphylococcus aureus* (MRSA) bacteremia continues to represent a challenge in clinical treatment. Telavancin, a lipoglycopeptide with multiple mechanisms of action, has been proposed as a treatment option in these cases.

**Figure:**
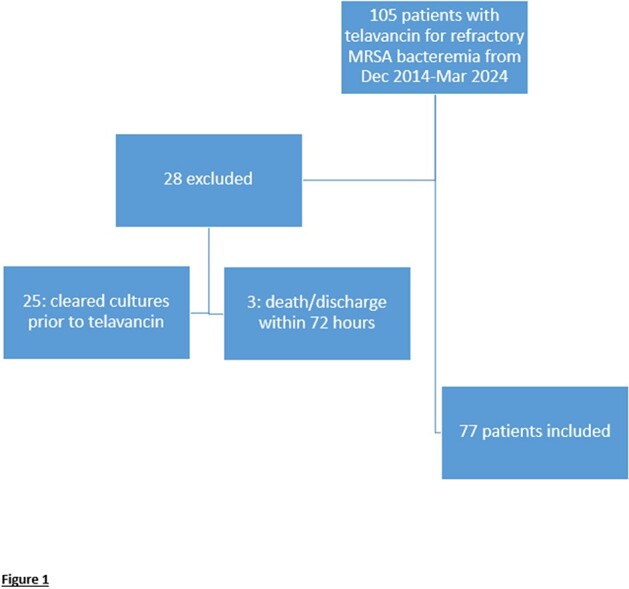

**Methods:**

This was a single-center, retrospective chart review of adult inpatients with refractory MRSA bacteremia (defined as > 72 hours of bacteremia following the start of daptomycin or vancomycin) who were treated with telavancin from December 2014 through March 2024. Patients who died or were discharged within 72 hours of starting telavancin or whose blood cultures cleared prior to the start of telavancin were excluded. More details can be seen in figure 1. The primary outcome was clinical success, defined as blood culture clearance on telavancin monotherapy. Secondary outcomes included length of time to blood culture clearance following telavancin initiation, recurrence of bacteremia within 30 days of clearance, and 30- and 90-day mortality from the time of telavancin initiation.

**Figure:**
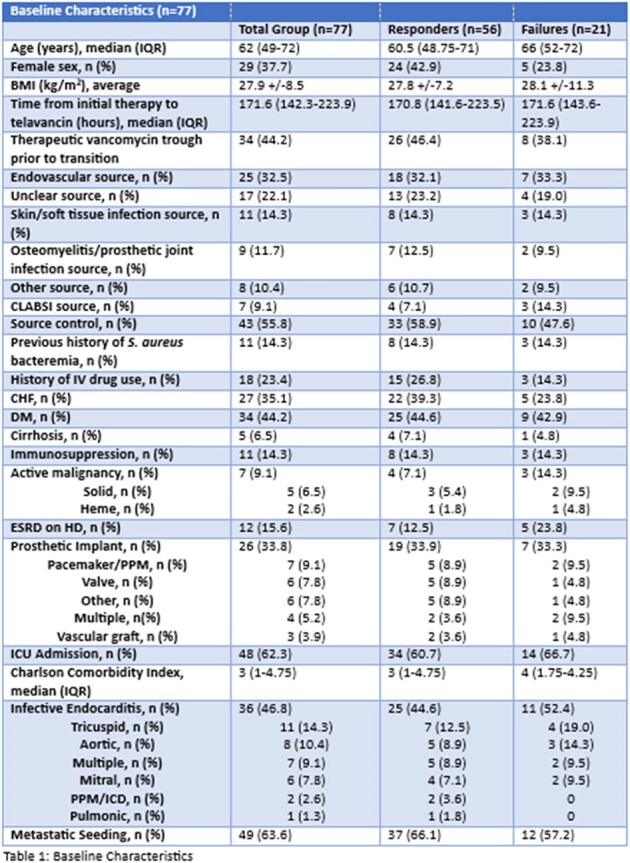

**Results:**

Of 105 patients who received telavancin, 77 met inclusion criteria. Of the patients who received telavancin, 56 (72.7%) met the primary outcome, and cleared the refractory MRSA bacteremia with telavancin monotherapy. Patients who failed telavancin included 11 who did not clear blood cultures before death, 2 who cleared on telavancin plus an additional agent, and 8 who cleared after switching from telavancin to another salvage regimen. Minimum inhibitory concentrations (MICs) ranged from ≤0.5 to 2 for vancomycin, 0.094 to 2 for daptomycin, and 0.016 to 0.38 for telavancin. Other baseline characteristics can be seen in table 1. No significant differences in baseline characteristics were seen between groups. The median time to culture clearance among the clinical success group was 57 hours. Only 1.8% of these patients had recurrent bacteremia within 30 days of telavancin initiation. At 30 days from telavancin initiation, overall mortality was 21.4%, and at 90 days, 23.2%.

**Figure:**
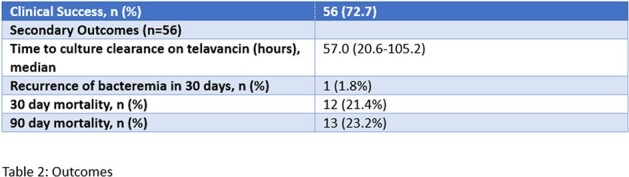

**Conclusion:**

In patients with refractory MRSA bacteremia, telavancin may be a viable monotherapy salvage regimen, with mortality rates similar to those previously reported for all patients with MRSA bacteremia.

**Disclosures:**

**All Authors**: No reported disclosures

